# Fever from Bad Milk

**Published:** 1884-08

**Authors:** 


### FEVER FROM BAD MILK.

DURING the last few years rapid strides have been made in the investigation of the diseases that may be transmitted directly or indirectly from the lower animals to man. It seems to be proved beyond doubt that foot-and-mouth disease is communicable by immediate contagion, and also by the unboiled milk of cattle suffering from the epidemic, even when the udders and teats are not visibly affected. With equal certainty outbreaks of enteric fever and other specific diseases incidental to humanity have been traced to the use of milk, although it is a moot question whether the milk obtained from infected districts is charged with the poison during the process of secretion, or whether it becomes subsequently contaminated by admixture with impure water. At the present time there is a serious outbreak of enteric fever in Dundee, which appears clearly traceable to the milk-supply of the locality invaded.—[*Lancet.*

